# (1*E*,2*E*)-1,2-Bis(2,3-dihydro-1*H*-inden-1-yl­idene)hydrazine

**DOI:** 10.1107/S160053681203560X

**Published:** 2012-08-23

**Authors:** Wolfgang Imhof, Joachim Wunderle

**Affiliations:** a Institute of Integrated Natural Sciences, Universitαtsstr. 1, 56070 Koblenz, Germany; b Institute of Inorganic and Analytical Chemistry, Friedrich-Schiller-University Jena, Humboldtstr. 8, 07743 Jena, Germany

## Abstract

In the title compound, C_18_H_16_N_2_, there are two independent half-mol­ecules (*A* and *B*) in the asymmetric unit, each mol­ecule being completed by an inversion center situated in the mid-point of the central N—N bond. The mol­ecules themselves therefore are essentially planar with r.m.s. deviations of 0.015 (1) and 0.020 (1) Å, respectively. In the crystal, mol­ecules are connected *via* C—H⋯π inter­actions in which only type *B* mol­ecules are donors, while both *A* and *B* mol­ecules act as acceptors. As a result, type *B* mol­ecules are linked into infinite chains along *b*, which are inter­connected by molecules of type *A*.

## Related literature

For structural and physical properties of indanone-derived azines, see: Choytun *et al.* (2004 ▶). For the reactivity of azines towards Fe_2_(CO)_9_, see: Dönnecke *et al.* (2004*a* ▶,*b* ▶); Wu *et al.* (2006 ▶).
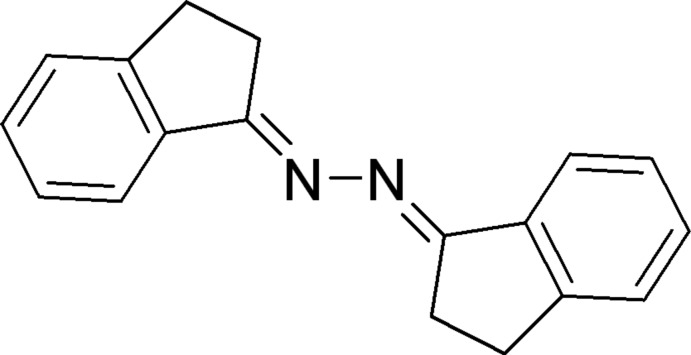


## Experimental

### 

#### Crystal data

C_18_H_16_N_2_
*M* *_r_* = 260.33
Triclinic, 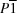

*a* = 5.1161 (10) Å
*b* = 11.877 (2) Å
*c* = 12.245 (2) Å
α = 109.59 (3)°
β = 99.93 (3)°
γ = 100.47 (3)°
*V* = 667.1 (2) Å^3^
*Z* = 2
Mo *K*α radiation
μ = 0.08 mm^−1^
*T* = 183 K
0.6 × 0.1 × 0.1 mm

#### Data collection

Nonius KappaCCD diffractometer
4726 measured reflections
2999 independent reflections
1901 reflections with *I* > 2σ(*I*)
*R*_int_ = 0.037

#### Refinement

*R*[*F* ^2^ > 2σ(*F* ^2^)] = 0.048
*wR*(*F* ^2^) = 0.114
*S* = 1.02
2999 reflections
181 parameters
H-atom parameters constrained
Δρ_max_ = 0.19 e Å^−3^
Δρ_min_ = −0.20 e Å^−3^

### 

Data collection: *COLLECT* (Nonius, 1998 ▶); cell refinement: *DENZO* (Otwinowski & Minor, 1997 ▶); data reduction: *DENZO*; program(s) used to solve structure: *SHELXS97* (Sheldrick, 2008 ▶); program(s) used to refine structure: *SHELXL97* (Sheldrick, 2008 ▶); molecular graphics: *XP* in *SHELXTL* (Sheldrick, 2008 ▶); software used to prepare material for publication: *publCIF* (Westrip, 2010 ▶).

## Supplementary Material

Crystal structure: contains datablock(s) I, global. DOI: 10.1107/S160053681203560X/bg2471sup1.cif

Structure factors: contains datablock(s) I. DOI: 10.1107/S160053681203560X/bg2471Isup2.hkl

Supplementary material file. DOI: 10.1107/S160053681203560X/bg2471Isup3.mol

Supplementary material file. DOI: 10.1107/S160053681203560X/bg2471Isup4.mol

Supplementary material file. DOI: 10.1107/S160053681203560X/bg2471Isup5.cml

Additional supplementary materials:  crystallographic information; 3D view; checkCIF report

## Figures and Tables

**Table 1:** Hydrogen-bond geometry (Å, °) *Cg*1 and *Cg*2 are the centroids of the C1–C6 and C10–C15 rings, respectively.

*D*—H⋯*A*	*D*—H	H⋯*A*	*D*⋯*A*	*D*—H⋯*A*
C17—H17*B*⋯*Cg*1	0.99	2.76	3.687 (3)	157
C16—H16*B*⋯*Cg*2^i^	0.99	2.79	3.649 (2)	146

Symmetry code: (i) 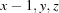.

**Fig. 1.**
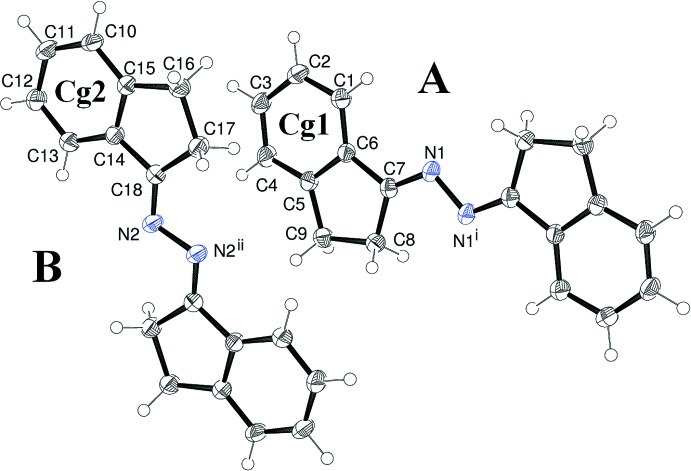
: Molecular structure of the independent molecules of the title compounds with displacement ellipsoids at the 50% probability level (i = -x, 1-y, 2 - z; ii = -x, - y, 1- z).

**Fig. 2.**
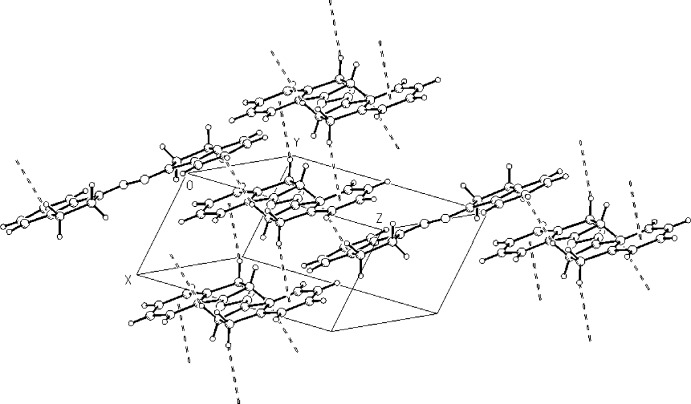
: Packing diagram of the title compound.

## supplementary crystallographic information

### Comment

In the course of a study concerning the reactivity of azines towards Fe_2_(CO)_9_ we became interested in the structual properties of several substituted aromatic azine derivatives (Dönnecke *et al.*, 2004*a*, 2004*b*). Azines derived from indanone derivatives have been investigated concerning their structural and physical properties due to the fact that some of them exhibit NLO properties (Choytun *et al.*, 2004).

In the asymmetric unit of the crystal structure two independent halves of the molecules of the title compound, C_18_H_16_N_2_, are observed (fIG. 1). Each fragment is expanded to a complete molecule by crystallographic inversion centers that are situated in the middle of the central N—N bonds of both molecules. The molecules themselves therefore are essentially planar with Rms deviations of 0.015 (1) (molecule A: N1, C1 to C9) and 0.020 (1) (molecule B: N2, C10 to C18) Å, respectively. The central N—N bonds are 1.412 (2) (N1—N1^i^) and 1.415 (3) (N2—N2^ii^) Å (i = -*x*, 1-*y*, 2- *z*; ii = -*x*, - *y*, 1- *z*). The planes of the two molecules form an angle of 5.77 (7)° with respect to each other. In the crystal structure molecules are connected *via* C—H···π interactions (Cg···Cg distances: 2.76 and 2.79 Å). Molecules B are linked by mutual C—H···π contacts in which the molecules act as hydrogen bond donors and acceptors resulting in infinite chains. These chains are interconnected by molecules A which only act as acceptor sites for C—H···π interactions (Figure 2).

### Experimental

2,3-Dihydro-1*H*-inden-1-one (2 g, 1.8 ml, 15.15 mmol) is dissolved in 30 ml ethanol and mixed with hydrazine hydrate (379 mg, 0.37 ml, 7.58 mmol) in the presence of a catalytic amount of *p*-toluene sulfonic acid. The resulting orange colored mixture is stirred at room temperature for 3 h. Evaporation of the solvent at room temperature for 3 d leads to the formation of the crystalline title compound (yield: 78%).

### Refinement

Hydrogen atoms have been placed in idealized positions and were refined using the riding model approximation with C—H distances of 0.95 and 0.99 Å for aromatic and methylene groups, respectively, with *U*_iso_(H) = 1.2 *U*_eq_(C).

### Crystal data

**Table:**

C_18_H_16_N_2_	*Z* = 2
*M**_r_* = 260.33	*F*(000) = 276
Triclinic, *P*1	*D*_x_ = 1.296 Mg m^−^^3^
Hall symbol: -P 1	Mo *K*α radiation, λ = 0.71073 Å
*a* = 5.1161 (10) Å	Cell parameters from 4726 reflections
*b* = 11.877 (2) Å	θ = 1.8–27.5°
*c* = 12.245 (2) Å	µ = 0.08 mm^−^^1^
α = 109.59 (3)°	*T* = 183 K
β = 99.93 (3)°	Quader, yellow
γ = 100.47 (3)°	0.6 × 0.1 × 0.1 mm
*V* = 667.1 (2) Å^3^	

### Data collection

**Table:**

Nonius KappaCCD diffractometer	1901 reflections with *I* > 2σ(*I*)
Radiation source: fine-focus sealed tube	*R*_int_ = 0.037
Graphite monochromator	θ_max_ = 27.5°, θ_min_ = 1.8°
phi–scan, ω–scan	*h* = −6→6
4726 measured reflections	*k* = −14→15
2999 independent reflections	*l* = −15→15

### Refinement

**Table:**

Refinement on *F*^2^	Primary atom site location: structure-invariant direct methods
Least-squares matrix: full	Secondary atom site location: difference Fourier map
*R*[*F*^2^ > 2σ(*F*^2^)] = 0.048	Hydrogen site location: inferred from neighbouring sites
*wR*(*F*^2^) = 0.114	H-atom parameters constrained
*S* = 1.02	*w* = 1/[σ^2^(*F*_o_^2^) + (0.050*P*)^2^] where *P* = (*F*_o_^2^ + 2*F*_c_^2^)/3
2999 reflections	(Δ/σ)_max_ < 0.001
181 parameters	Δρ_max_ = 0.19 e Å^−^^3^
0 restraints	Δρ_min_ = −0.20 e Å^−^^3^

### Special details

**Table:**

Geometry. All e.s.d.'s (except the e.s.d. in the dihedral angle between two l.s. planes) are estimated using the full covariance matrix. The cell e.s.d.'s are taken into account individually in the estimation of e.s.d.'s in distances, angles and torsion angles; correlations between e.s.d.'s in cell parameters are only used when they are defined by crystal symmetry. An approximate (isotropic) treatment of cell e.s.d.'s is used for estimating e.s.d.'s involving l.s. planes.
Refinement. Refinement of *F*^2^ against ALL reflections. The weighted *R*-factor *wR* and goodness of fit *S* are based on *F*^2^, conventional *R*-factors *R* are based on *F*, with *F* set to zero for negative *F*^2^. The threshold expression of *F*^2^ > σ(*F*^2^) is used only for calculating *R*-factors(gt) *etc*. and is not relevant to the choice of reflections for refinement. *R*-factors based on *F*^2^ are statistically about twice as large as those based on *F*, and *R*- factors based on ALL data will be even larger.

### Fractional atomic coordinates and isotropic or equivalent isotropic displacement parameters (Å^2^)

**Table:**

	*x*	*y*	*z*	*U*_iso_*/*U*_eq_	
N1	0.0535 (2)	0.51077 (11)	0.95393 (11)	0.0280 (3)	
C1	0.3116 (3)	0.50842 (14)	0.74721 (14)	0.0304 (4)	
H1	0.2287	0.5754	0.7683	0.036*	
C2	0.4487 (3)	0.49064 (14)	0.65753 (14)	0.0338 (4)	
H2	0.4614	0.5461	0.6168	0.041*	
N2	0.0565 (3)	−0.00657 (12)	0.45007 (11)	0.0319 (3)	
C3	0.5684 (3)	0.39184 (14)	0.62645 (14)	0.0326 (4)	
H3	0.6627	0.3807	0.5648	0.039*	
C4	0.5518 (3)	0.30961 (14)	0.68430 (14)	0.0309 (4)	
H4	0.6330	0.2421	0.6624	0.037*	
C5	0.4150 (3)	0.32704 (13)	0.77458 (13)	0.0243 (4)	
C6	0.2971 (3)	0.42640 (13)	0.80617 (13)	0.0238 (3)	
C7	0.1704 (3)	0.42645 (13)	0.90464 (13)	0.0237 (3)	
C8	0.2091 (3)	0.31658 (13)	0.93521 (14)	0.0276 (4)	
H8B	0.0291	0.2597	0.9222	0.033*	
H8A	0.3110	0.3439	1.0199	0.033*	
C9	0.3745 (3)	0.25193 (13)	0.85068 (14)	0.0291 (4)	
H9B	0.5534	0.2519	0.8969	0.035*	
H9A	0.2712	0.1654	0.8005	0.035*	
C10	0.1242 (3)	0.20212 (14)	0.17948 (13)	0.0314 (4)	
H10	0.0774	0.2678	0.1594	0.038*	
C11	0.2587 (3)	0.12604 (15)	0.11147 (15)	0.0365 (4)	
H11	0.3040	0.1397	0.0441	0.044*	
C12	0.3292 (3)	0.02931 (15)	0.13998 (14)	0.0356 (4)	
H12	0.4231	−0.0217	0.0924	0.043*	
C13	0.2633 (3)	0.00749 (14)	0.23669 (14)	0.0302 (4)	
H13	0.3104	−0.0583	0.2565	0.036*	
C14	0.1258 (3)	0.08405 (13)	0.30495 (13)	0.0250 (3)	
C15	0.0581 (3)	0.18161 (13)	0.27746 (13)	0.0259 (4)	
C16	−0.0860 (3)	0.25116 (13)	0.36545 (14)	0.0302 (4)	
H16B	−0.2718	0.2490	0.3237	0.036*	
H16A	0.0203	0.3385	0.4089	0.036*	
C17	−0.1032 (3)	0.18309 (14)	0.45227 (14)	0.0289 (4)	
H17B	−0.0041	0.2396	0.5352	0.035*	
H17A	−0.2971	0.1508	0.4506	0.035*	
C18	0.0289 (3)	0.07840 (13)	0.40943 (13)	0.0250 (3)	

### Atomic displacement parameters (Å^2^)

**Table:**

	*U*^11^	*U*^22^	*U*^33^	*U*^12^	*U*^13^	*U*^23^
N1	0.0332 (7)	0.0306 (7)	0.0251 (7)	0.0124 (6)	0.0151 (6)	0.0108 (6)
C1	0.0360 (9)	0.0302 (9)	0.0310 (9)	0.0135 (7)	0.0142 (7)	0.0136 (7)
C2	0.0429 (10)	0.0356 (9)	0.0322 (9)	0.0135 (8)	0.0172 (8)	0.0190 (8)
N2	0.0459 (8)	0.0310 (7)	0.0301 (8)	0.0157 (6)	0.0198 (6)	0.0174 (6)
C3	0.0365 (9)	0.0392 (9)	0.0297 (9)	0.0143 (7)	0.0183 (8)	0.0150 (8)
C4	0.0328 (9)	0.0349 (9)	0.0317 (9)	0.0171 (7)	0.0162 (7)	0.0126 (8)
C5	0.0221 (7)	0.0268 (8)	0.0242 (8)	0.0068 (6)	0.0072 (7)	0.0091 (7)
C6	0.0251 (8)	0.0236 (7)	0.0223 (8)	0.0061 (6)	0.0079 (6)	0.0073 (6)
C7	0.0223 (7)	0.0238 (8)	0.0233 (8)	0.0059 (6)	0.0063 (6)	0.0066 (6)
C8	0.0298 (8)	0.0270 (8)	0.0289 (9)	0.0080 (7)	0.0120 (7)	0.0115 (7)
C9	0.0311 (8)	0.0280 (8)	0.0325 (9)	0.0116 (7)	0.0123 (7)	0.0125 (7)
C10	0.0423 (9)	0.0325 (9)	0.0300 (9)	0.0151 (7)	0.0142 (8)	0.0195 (8)
C11	0.0509 (10)	0.0400 (9)	0.0322 (9)	0.0170 (8)	0.0226 (8)	0.0219 (8)
C12	0.0476 (10)	0.0350 (9)	0.0333 (10)	0.0187 (8)	0.0227 (8)	0.0141 (8)
C13	0.0377 (9)	0.0265 (8)	0.0310 (9)	0.0112 (7)	0.0137 (7)	0.0126 (7)
C14	0.0261 (8)	0.0256 (8)	0.0229 (8)	0.0052 (6)	0.0064 (6)	0.0092 (7)
C15	0.0284 (8)	0.0268 (8)	0.0243 (8)	0.0078 (6)	0.0084 (7)	0.0106 (7)
C16	0.0368 (9)	0.0296 (8)	0.0318 (9)	0.0154 (7)	0.0131 (7)	0.0152 (7)
C17	0.0321 (8)	0.0325 (8)	0.0271 (8)	0.0126 (7)	0.0110 (7)	0.0136 (7)
C18	0.0267 (8)	0.0248 (8)	0.0242 (8)	0.0070 (6)	0.0070 (7)	0.0096 (7)

### Geometric parameters (Å, º)

**Table:**

N1—C7	1.2895 (18)	C9—H9B	0.9900
N1—N1^i^	1.412 (2)	C9—H9A	0.9900
C1—C2	1.381 (2)	C10—C11	1.379 (2)
C1—C6	1.393 (2)	C10—C15	1.385 (2)
C1—H1	0.9500	C10—H10	0.9500
C2—C3	1.393 (2)	C11—C12	1.396 (2)
C2—H2	0.9500	C11—H11	0.9500
N2—C18	1.2847 (19)	C12—C13	1.377 (2)
N2—N2^ii^	1.415 (2)	C12—H12	0.9500
C3—C4	1.385 (2)	C13—C14	1.395 (2)
C3—H3	0.9500	C13—H13	0.9500
C4—C5	1.386 (2)	C14—C15	1.394 (2)
C4—H4	0.9500	C14—C18	1.467 (2)
C5—C6	1.393 (2)	C15—C16	1.512 (2)
C5—C9	1.505 (2)	C16—C17	1.541 (2)
C6—C7	1.464 (2)	C16—H16B	0.9900
C7—C8	1.509 (2)	C16—H16A	0.9900
C8—C9	1.544 (2)	C17—C18	1.506 (2)
C8—H8B	0.9900	C17—H17B	0.9900
C8—H8A	0.9900	C17—H17A	0.9900
			
C7—N1—N1^i^	111.59 (15)	H9B—C9—H9A	108.9
C2—C1—C6	118.82 (14)	C11—C10—C15	119.20 (14)
C2—C1—H1	120.6	C11—C10—H10	120.4
C6—C1—H1	120.6	C15—C10—H10	120.4
C1—C2—C3	120.35 (15)	C10—C11—C12	121.08 (15)
C1—C2—H2	119.8	C10—C11—H11	119.5
C3—C2—H2	119.8	C12—C11—H11	119.5
C18—N2—N2^ii^	111.52 (15)	C13—C12—C11	120.29 (15)
C4—C3—C2	120.84 (14)	C13—C12—H12	119.9
C4—C3—H3	119.6	C11—C12—H12	119.9
C2—C3—H3	119.6	C12—C13—C14	118.55 (15)
C5—C4—C3	119.13 (14)	C12—C13—H13	120.7
C5—C4—H4	120.4	C14—C13—H13	120.7
C3—C4—H4	120.4	C13—C14—C15	121.22 (14)
C4—C5—C6	119.98 (14)	C13—C14—C18	128.93 (15)
C4—C5—C9	128.68 (13)	C15—C14—C18	109.84 (13)
C6—C5—C9	111.34 (12)	C10—C15—C14	119.64 (14)
C1—C6—C5	120.89 (14)	C10—C15—C16	129.42 (14)
C1—C6—C7	129.37 (14)	C14—C15—C16	110.94 (13)
C5—C6—C7	109.75 (13)	C15—C16—C17	104.84 (12)
N1—C7—C6	122.75 (14)	C15—C16—H16B	110.8
N1—C7—C8	128.85 (14)	C17—C16—H16B	110.8
C6—C7—C8	108.39 (12)	C15—C16—H16A	110.8
C7—C8—C9	105.79 (12)	C17—C16—H16A	110.8
C7—C8—H8B	110.6	H16B—C16—H16A	108.9
C9—C8—H8B	110.6	C18—C17—C16	105.91 (12)
C7—C8—H8A	110.6	C18—C17—H17B	110.6
C9—C8—H8A	110.6	C16—C17—H17B	110.6
H8B—C8—H8A	108.7	C18—C17—H17A	110.6
C5—C9—C8	104.71 (12)	C16—C17—H17A	110.6
C5—C9—H9B	110.8	H17B—C17—H17A	108.7
C8—C9—H9B	110.8	N2—C18—C14	122.08 (14)
C5—C9—H9A	110.8	N2—C18—C17	129.46 (14)
C8—C9—H9A	110.8	C14—C18—C17	108.45 (13)

Symmetry codes: (i) −*x*, −*y*+1, −*z*+2; (ii) −*x*, −*y*, −*z*+1.

### Hydrogen-bond geometry (Å, º)

Cg1 and Cg2 are the centroids of the C1–C6 and C10–C15 rings, respectively.

**Table:**

*D*—H···*A*	*D*—H	H···*A*	*D*···*A*	*D*—H···*A*
C17—H17*B*···*Cg*1	0.99	2.76	3.687 (3)	157
C16—H16*B*···*Cg*2^iii^	0.99	2.79	3.649 (2)	146

Symmetry code: (iii) *x*−1, *y*, *z*.
